# Variation in pulmonary function tests among children with sickle cell anemia: a systematic review and meta-analysis

**DOI:** 10.11604/pamj.2021.39.140.28755

**Published:** 2021-06-18

**Authors:** Amar Taksande, Patel Zeeshan Jameel, Divya Pujari, Bharati Taksande, Revat Meshram

**Affiliations:** 1 Department of Paediatrics, Jawaharlal Nehru Medical College, Datta Meghe Institute of Medical Sciences, Sawangi Meghe, Wardha, Maharashtra State, India,; 2 Department of Paediatrics, Bai Jerbai Wadia Hospital for Children, Parel, Mumbai, Maharashtra, India,; 3 Department of Medicine, Mahatma Gandhi Institute of Medical Sciences (MGIMS), Sewagram, Wardha, Maharashtra State, India

**Keywords:** Sickle cell anaemia, pulmonary function tests, spirometry

## Abstract

**Introduction:**

the spectrum of pulmonary complications in sickle cell anemia (SCA) comprises mainly of acute chest syndrome (ACS), pulmonary hypertension (PH) and airway hyper-responsiveness (AHR). This study was conducted to examine the abnormalities in pulmonary function tests (PFTs) seen in children with SCA.

**Methods:**

electronic databases (Cochrane library, PubMed, EMBASE, Scopus, Web of Science) were used as data sources. Two authors independently reviewed studies. All case-control studies with PFT performed in patients with SCA and normal controls were reviewed. Pulmonary functions were assessed with the help of spirometry, lung volume and gas diffusion findings.

**Results:**

nine studies with 788 SCA children and 1101 controls were analyzed. For all studies, the pooled mean difference for forced expiratory volume in 1 second (FEV1), forced vital capacity (FVC), FEV1/FVC ratio, peak expiratory flow rate (PEFR), total lung capacity (TLC) and carbon mono-oxide diffusing capacity (DLCO) were -12.67, (95% CI: -15.41,-9.94), -11.69, (95% CI: -14.24, -9.14), -1.90, (95% CI: -4.32, 0.52), -3.36 (95% CI: -6.69, -0.02), -7.35, (95% CI: -14.97, -0.27) and -4.68, (95% CI -20.64, -11.29) respectively. FEV1 and FVC and were the only parameters found to be significantly decreased.

**Conclusion:**

sickle cell anemia was associated with lower FEV1 and FVC, thus, supporting the role of routine monitoring for the progression of lung function decline in children with SCA with ACS. We recommend routine screening and lung function monitoring for early recognition of pulmonary function decline.

## Introduction

Sickle-cell Anemia (SCA) is a monogenic, hematological, multi-system disease characterized by repeated episodes of acute disease exacerbations resulting in multi-organ damage. Globally, it has been projected that the number of individuals with SCA are set to increase dramatically from 305,800 in 2010 to 404,200 by 2050 [1]. The mortality rate among children is estimated to be 0.64 per 100 years of child observation with the maximal rate being among the children belonging to African ethnicity (7.3 per 100 years of observation) [2]. Pulmonary disease is a leading cause of mortality and morbidity among patients with SCA and is the second most common underlying pathology requiring hospital admission [3]. The disease spectrum of pulmonary complications in SCA comprises of acute chest syndrome (ACS), pulmonary hypertension (PH), lower airway obstruction and airway hyper-responsiveness (AHR). Frequent episodes of ACS are being increasingly recognized as a major risk factor for sickle-cell associated chronic lung disease [4]. Acute chest syndrome depicts a morbid chain of reaction of pulmonary infarction, inflammation and atelectasis resulting in ventilation-perfusion mismatch and acutely increased pressure in pulmonary artery [5]. In addition, these changes in lung parenchyma and its vasculature along with hemolysis contribute to the development of PH in SCA. The prevalence of pulmonary hypertension (PH) among children with SCA is approximately 1 in every 5 children with SCA [6] and is a major risk factor associated with mortality [6-8]. Airway hyper-responsiveness (AHR) was first evaluated by Leong *et al* among children with SCA [9]. Current literature and evidence have brought forth the fact that there may be significant overlap in the pathophysiology of pulmonary insults in asthma and SCA [10,11]. The prevalence of asthma in children with SCA has been reported to be between 16.8% to 27% in studies respectively [12,13]. As asthma is a modifiable risk factor, the morbidity associated with it may be decreased substantially by its early diagnosis and effective control. Pulmonary function tests (PFTs) helps to confirm the diagnosis and hence, significantly improve the clinical management of asthma [13]. Current evidence support the opinion that existence of pulmonary disease in children with SCA significantly increases the morbidity. However, the pattern of abnormal lung function in SCA has been disputed. Herein, we discuss the first systematic review and meta-analysis of studies evaluating the PFT in children with SCA in comparison to children without SCA. In addition, we have tried to determine if there is an association of hydroxyurea therapy with PFT parameters among children with SCA.

## Methods

PRISMA (Preferred Reporting Items for Systematic reviews and Meta-analyses) guideline was used to report this study [14]. The protocol for this systematic review and meta-analysis was registered at the International Prospective Register of Systematic Reviews (PROSPERO # CRD42020206113).

**Search strategy for identifying relevant studies:** the search strategy was implemented in two stages:

**Bibliographic database search:** electronic databases (Cochrane library, PubMed, EMBASE, Scopus, Web of Science) were used as data sources. Search was restricted to English language publications involving human subjects, but not restricted by date or publication type. The last electronic search was carried out on 20^th^ August, 2020. The search strategy included the following terms: (“respiratory function tests”[MeSH Terms] OR (“respiratory”[All Fields] AND “function”[all fields] AND “tests”[all fields]) OR “respiratory function tests”[all fields] OR (“pulmonary”[all fields] AND “function”[all felds] AND “tests”[all fields]) OR “pulmonary function tests”[all fields]) AND (“anemia, sickle cell”[MeSH Terms] OR (“anemia”[all fields] AND “sickle”[all fields] AND “cell”[all fields]) OR “sickle cell anemia”[all fields] OR (“sickle”[all fields] AND “cell”[all fields] AND “disease”[all fields]) OR “sickle cell disease”[all fields]).

**Searching other sources:** we conducted manual searches which consisted of scanning the reference lists of eligible papers and other relevant review articles, specialist journals and conference proceedings. All studies were imported to the literature management software Endnote X7 to eliminate duplicated records.

**Eligibility criteria for considering studies to include in the review:** studies considered in this meta-analysis were case-control studies about any alteration in pulmonary functions in sickle cell anemia children.

**Inclusion criteria:** i) Studies meeting the defined PECO-S criteria were included [population-children (age range: 3-18 years) with SCA, exposure-sickle cell anemia (SCA), comparator-children without SCA, outcomes-pulmonary function parameters, study design-case-control or comparative study]. ii) Studies where main outcomes evaluated were forced expiratory volume in 1 second (FEV1), forced vital capacity (FVC), FEV1/FVC ratio and total lung capacity (TLC). FEV1, FVC and TLC were expressed as percent (%) predicted. FEV1/FVC ratio was expressed as an absolute value.

**Exclusion criteria:** i) Studies with no control group; ii) Studies not performed in human participants; iii) Case series, reviews, letters, commentaries and editorials, and iv) Studies with insufficient data, abstracts, adult studies, conference abstracts and duplicate publications.

**Selection of studies for inclusion in the review:** two authors (PZJ and DP) independently conducted a preliminary screening of studies by reading titles and abstracts. After screening titles and abstracts, the full texts of potentially relevant articles were downloaded. These investigators further independently assessed the full text of each study for eligibility and consensually retained studies were included. Disagreements when existing were resolved by a third author (AT). Studies were selected if they met the inclusion criteria. We used a screening guide to ensure that all review authors reliably applied the selection criteria. Agreement was measured using the kappa (κ) statistic [15]. Data in studies, using median and standard error as a measure of dispersion, was converted to mean and standard deviation [16].

**Data extraction and management:** data extraction and quality control was independently done by two reviewers (PZJ and DP). A third reviewer (AT) was involved if conflict occurred. A standard data extraction form was used to retrieve relevant information and data from each study included in the analysis. Two review authors (PZJ and DP) participated in data extraction independently. PZJ and DP extracted data which included general information (authors, year, and country), design of the study, history of ACS and/or AHR and various parameters of pulmonary function test. Outcome measures included assessment of lung function by FEV1, FVC, FEV1/ FVC, PEFR, TLC and DLCO with their corresponding SD, SE or 95% CI. We focused on spirometry, lung volume and gas diffusion findings. Because FEV1, FVC, FEV1/FVC and TLC are the basic parameters needed to identify a functional deficit (i.e. restriction or obstruction), we primarily focused on these for our analysis. We looked at each endpoint separately and in combination to determine the variation in the various parameters of PFT in each study. We considered both the statistical and clinical significance of lung function results.

**Statistical analysis:** the mean differences of various parameters of pulmonary function test results were synthesized using a random-effects meta-analysis between the SCA children and control included FEV1 (unit in predictive percentage), FVC (unit in predictive percentage), FEV1/FVC (unit in the original ratio), PEFR (unit in predictive percentage), TLC (unit in predictive percentage) and DLCO (unit in predictive percentage). The software Review Manager 5.3 (The Nordic Cochrane Centre, Copenhagen, Denmark) was used to conduct meta-analyses for the outcome measures, reported as the mean difference with 95% confidence interval (CI). Heterogeneity was defined as low, moderate, or high according to I^2^ values (25%, 50%, or 75%, respectively). A forest plot was used to display the results of the meta-analysis. Data analysis was performed using the fixed-effects model when the results showed low heterogeneity (I^2^ ≤25%) and the random-effects model when the results showed moderate or high heterogeneity (I^2^ >25%). Publication bias was assessed by viewing the symmetry of the funnel plot. We then investigated reasons for heterogeneity by fitting meta-regression models. High-resolution forest plots, with random effects, were separately created.

## Results

**Figure 1:**
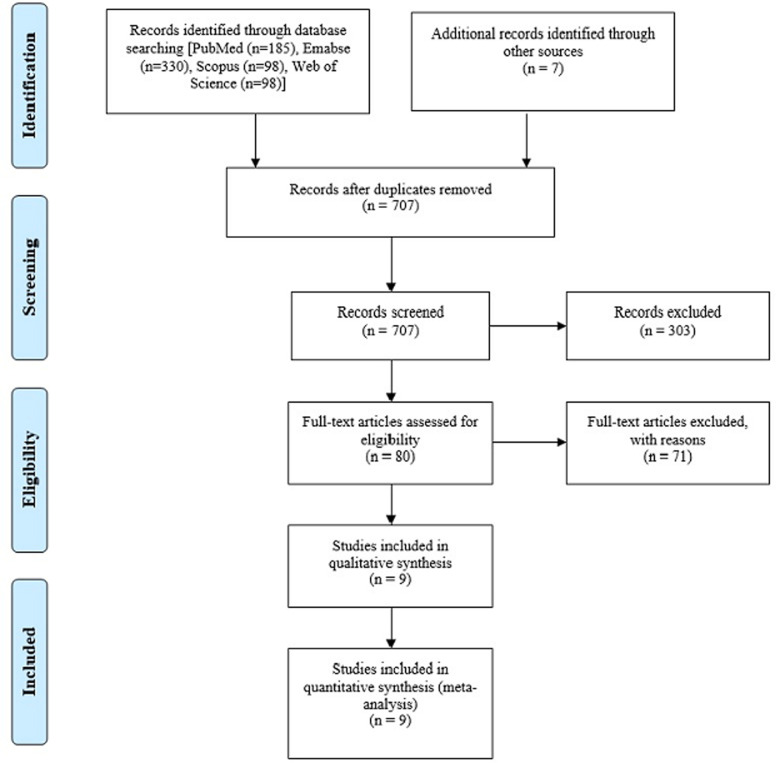
PRISMA flow chart diagram describing Process of identification and selection of studies for inclusion in the review

**Literature search:** initially, a total of 707 articles were identified. After elimination of duplicates, screening titles and abstracts, 303 papers were found completely irrelevant and excluded. Agreement between investigators on abstract selection was high (κ =0.90, p <0.001). Full texts of the remaining 80 studies were scrutinized for eligibility, among which 71 studies were excluded. There was no disagreement between investigators for full-text selection. Overall, case-control studies satisfied the eligibility criteria and hence, were included in the meta-analysis (Figure 1).

**Basic characteristics of the included studies:** after the application of the exclusion criteria, nine studies with a combined study population of 1889 were included in this meta-analysis. These included 788 children with SCA and 1101 normal controls. However, in the absence of controls for UK subgroup of SCA children in the study by Arigliani *et al*. [17], they are excluded from meta-analysis. The characteristics of the included studies are shown in Table 1. Studies were conducted in six countries, and they were published between 1993 and 2020 (Table 1). The sample size of individual studies ranged from 38 to 518 participants. Participant's age ranged from 3 to 18 years of age and included both male and female children. Although spirometry is an effort-dependent test, all study explicitly stated that they had performed lung function tests in accordance with international guidelines set forth jointly by the American Thoracic Society (ATS) and the European Respiratory Society (ERS), thus, facilitating spirometry maneuver standardization (Table 2).

**Table 1:** baseline characteristics of the included studies in the meta-analysis

S.No.	Author	Year of publication	Country	Study design	Population size			Age (range mean±SD)		Sex (M:F)	
					Total	Cases	Controls	Cases	Controls	Cases	Controls
1	Pianosi P *et al*. (HbSS group)	1993	Canada	Case control	33	11	22	14±4.1	12±3.5	3:8	9:13
	Pianosi P *et al*. [18] (ACS group)	1993	Canada	Case control	32	10	22	12±3.1	12±3.5	7:3	9:13
2	Hijazi Z *et al*. (HbSS)	2005	Kuwait	Case control	38	21	17	12.1±4	10.8±12.6	NM	NM
3	Sylvester KP *et al*.	2007	UK	Case control	48	24	24	11(8-16)	11(7-16)	14:10	14:10
4	Wedderburn CJ *et al*.	2014	UK	Case control	50	25	25	13.4(7.4-18.2)	13(7.4-18)	11:14	8:17
5	Purohit R *et al*.	2016	India	Case control	198	99	99	12.5±1.05	13.05±1.1	NM	NM
6	Lunt A *et al*. (Cohort 1)	2016	UK	Case control	73	47	26	8.8(3-13.1)	10.2(4-14.6)	21:26	7:19
	Lunt A *et al*. (Cohort 2)	2016	UK	Case control	69	45	24	10.2(4.3-16)	8.5(4-17.8)	19:26	8:16
7	Arigliani M *et al*. (Central Africa)	2019	Nigeria	Case control	489	112	377	11.3±3.3	9.5±1.6	66:46	207:170
8	Arigliani M *et al*.(Nigeria)	2019	Nigeria	Case control	518	154	364	11.4±3.2	10.4±2.4	83:71	189:175
	Arigliani M *et al*. (UK)	2019	United Kingdom	Case control	101	101	NA	11.7±2.7	NA	51:50	NA
9	Al-Biltagi M *et al*.	2020	Bahrain	Case control	262	139	123	11.9±4.1	12.1±4	84:55	71:51

NM=Not mentioned; NA=Not applicable; SD= Standard deviation

**Table 2:** screening methodology of the included studies

S.No.	Author	Spirometer/instrument used for PFT	Lung function	PFT measured (measured/residual/% predicted)	Criteria used	Inclusion of ACS in studies	Inclusion of children on hydroxyurea in studies	Inclusion of airway hyperresponsiveness (AHR) in studies
1	Pianosi P *et al*. (HbSS group)	Automated dry rolling seal spirometer (5000 IV: Gould inc., Oxnard, calif)	FEV1, FVC, FEV1/FVC, TLC, DLCO, FEF_25-75_, RV/TLC	% predicted	NR	No	No	No
	Pianosi P *et al*. (ACS group)	Automated dry rolling seal spirometer (5000 IV: Gould inc., Oxnard, calif)	FEV1, FVC, FEV1/FVC, TLC, DLCO, FEF_25-75_, RV/TLC	% predicted	NR	Yes (10/10)	No	No
2	Hijazi Z *et al*. (HbSS)	Constant volume- variable pressure body plethysmograph (Erich-jager master laboratory GmbH version 4.5, Hoechberg, Germany)	FEV1, FEV1/FVC, PEF, MEF 25/75, TLC	% predicted, measured	ATS	Yes (8/24)	No	No
3	Sylvester KP *et al*.	Dry rolling seal spirometer (Morgan TLC, Morgan medical, Rainham, UK)	FEV1, FVC, FEV1/FVC, PEFR, TLCpleth, VCpleth,	% predicted, residual	Values from study by Pellegrino *et al*.	Yes (4/24)	No	Yes (among controls - 7, 2 on medication)
4	Wedderburn CJ *et al*.	Whole body plethysmography with pnemutachograph based system (Jaeger masterscreen PFT, carefusion limited, Basingstoke, UK)	FEV1, VC, FEV1/VC, PEF, TLC, RV, DLCOc	% predicted	ATS/ETS	Yes (7/25)	Yes (7/25)	Yes (cases:8/25, controls: 4/25)
5	Purohit R *et al*.	Spirometer “spirolab 3” MIR010	FEV1, FVC, FEV1/FVC, PEFR	% predicted	ATS	Yes (11/99)	No	No
6	Lunt A *et al*. (Cohort 1)	Whole body plethysmography with pneumotachograph based system (Jaeger masterscreen PFT, carefusion limited, Basingstoke, UK))	FEV1, VC, FEV1/VC, TLC, RV, RV/TLC, FEF_25-75_	% predicted	ATS/ETS	Yes (10/47)	Yes (7/47)	Yes (cases: 9/47, controls: 2/26)
	Lunt A *et al*. (Cohort 2)	Whole body plethysmography with Pneumotachograph based system (Jaeger masterscreen PFT, carefusion limited, Basingstoke, UK))	FEV1, VC, FEV1/VC, TLC, RV, RV/TLC, FEF_25-75_	% predicted	ATS/ETS	Yes (12/45)	Yes (8/45)	Yes (cases: 3/45, controls: 4/24)
7	Arigliani M *et al*. (Central Africa)	Pony FX spirometer (COsmed, Rome, RM, Italy)	FEV1, FVC, zFEV1/FVC	% predicted	ERS	NR	NR	Yes (8/112)
8	Arigliani M *et al*. (Nigeria)	Easyon-PC ultrasonic flowmeter spirometer(ndd, Zurich Switzerland)	FEV1, FVC, FEV1/FVC	% predicted	ERS	Yes (33/154)	Yes (1/154)	Yes (10/154)
	Arigliani M *et al*. (UK)	Easyon-PC ultrasonic flowmeter spirometer(ndd, Zurich Switzerland)	FEV1, FVC, FEV1/FVC	% predicted	ERS	Yes (35/101)	Yes (48/101)	Yes (22/101)
9	Al-Biltagi M *et al*.	Jaeger masterscreen body/diffusion machine (vyaire medical inc, Chicago, Il, USA)	FEV1, FVC, FEV1/FVC, TLC, DLCO, RV	% predicted	ATS	Yes (85/139)	Yes (95/139)	No

FEV1: forced expiratory volume in 1 second; FEF_25-75_: forced expiratory flow between 25-75% of FVC; FVC: forced vital capacity; GLI: Global Lung Initiative; PEF: peak expiratory flow; PFT: pulmonary function tests; NR=Not reported; ERS=European respiratory society; ETS: European thoracic society; ATS=American Thoracic society

### Comparison of the sickle cell disease children and the normal children

**Figure 2:**
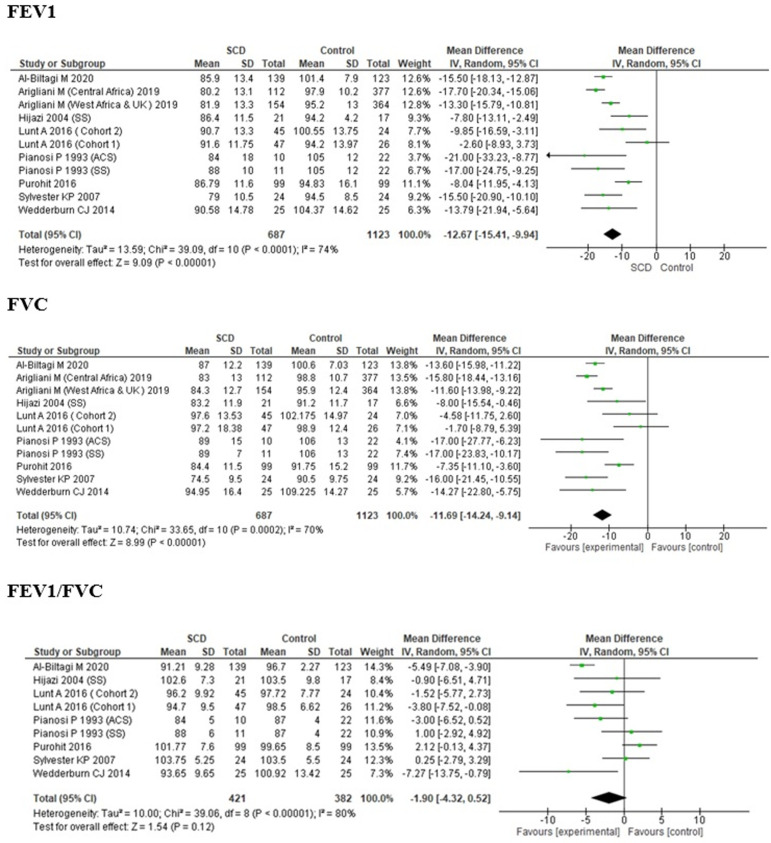
forest plot for FEV1 % predicted, FVC % predicted and FEV1/FVC ratio

**Forced expiratory volume in 1 second (FEV1):** the FEV1 % predictions of children with SCA were significantly lower than that of the controls [MD -12.67, (95% CI -15.41,-9.94), P<0.00001, I^2^=74%] among the 9 included studies (Figure 2).

**Forced vital capacity (FVC):** the FVC % predictions of children with SCA were significantly lower than that of the controls [MD -11.69, (95% CI -14.24, -9.14), P <0.00001, I^2^ =70%] among the 9 included studies (Figure 2).

**Forced expiratory volume in 1 second (FEV1)/Forced vital capacity (FVC) ratio:** the FEV1/FVC ratio of the children with SCA was not significantly lower than that of the controls [MD -1.90, (95% CI -4.32, 0.52), P=0.12, I^2^ =80%] among the 7 included studies (Figure 2).

**Figure 3:**
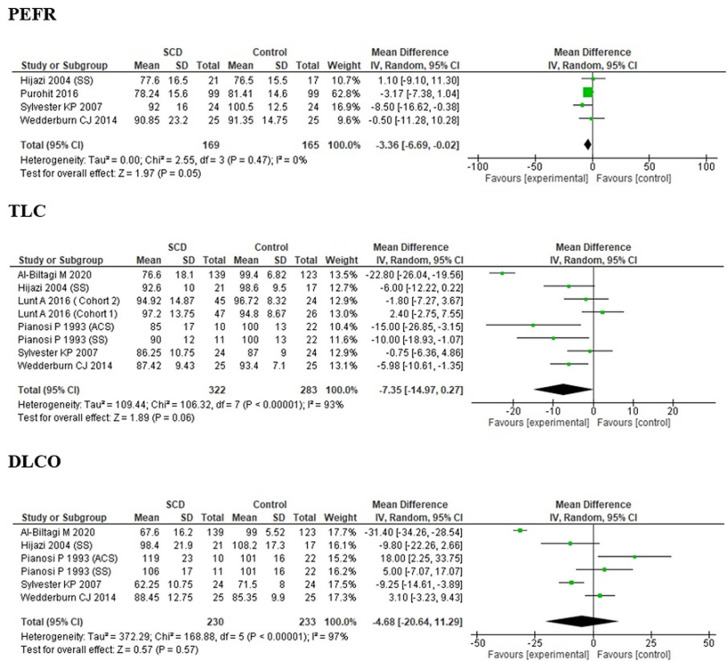
forest plot of PEFR, TLC and DLCO

**Peak expiratory flow rate (PEFR):** only four studies reporting PEFR of children with SCA in comparison to that of normal children as controls shows that the pooled estimates of PEFR for SCA children were lower than that for controls [MD -3.36, (95% CI -6.69, -0.02), P=0.05, I^2^=0%] (Figure 3).

**Total lung capacity (TLC):** the TLC of children with SCA cases was observed to be lower than that of the controls [MD -7.35, (95% CI -14.97, 0.27), P= 0.06, I^2^ =93%] among the 6 included studies (Figure 3).

**Carbon mono-oxide diffusing capacity (DLCO):** the DLCO of children with SCA was observed to be lower than that of control [MD -4.68, (95% CI -20.64, 11.29), P=0.57, I^2^=97%] (Figure 3).

**Figure 4:**
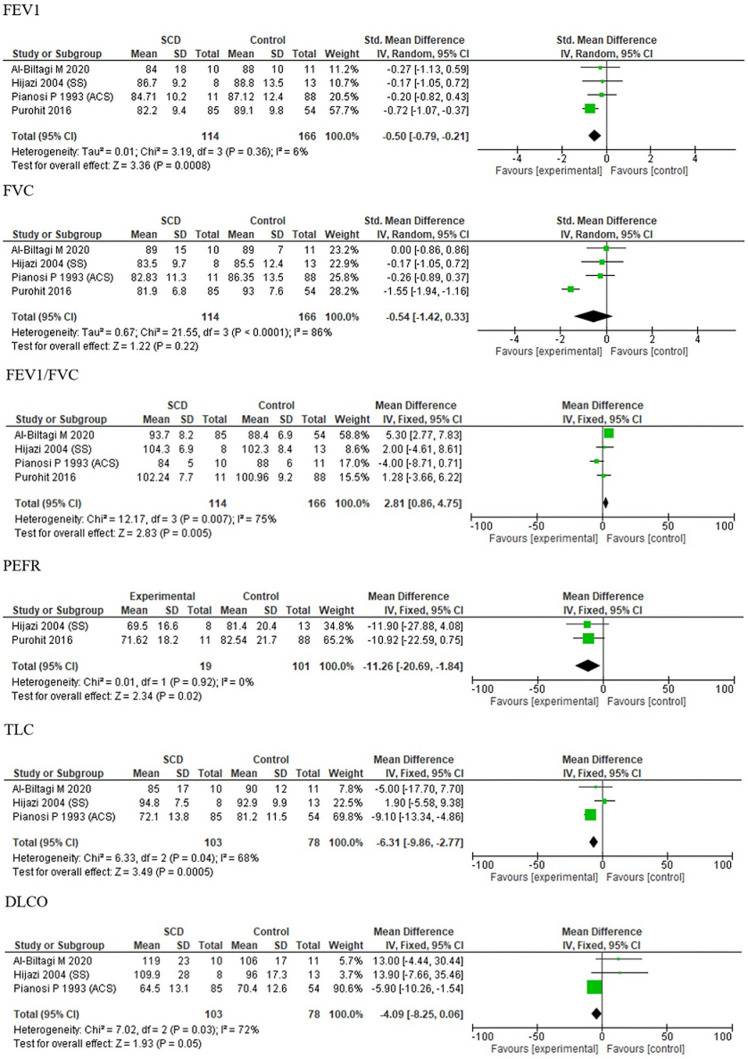
forest plot of forest plots of PFT parameters in children with SCA with history of ACS among the various studies

**Pulmonary function tests changes in studies including children with ACS:** four studies, which reported data on the basis of a history of ACS in SCA children were included. Pulmonary function test (PFT) parameters (FEV1, FVC, TLC, PEFR, and DLCO) of SCA children with ACS were observed to be lower than that of the control children. However, the ratio of FEV1/FVC was higher than the controls in three studies [18-21] while it was lower in one study [21]. The plots of pooled estimates for PFT parameters suggested no significant heterogeneity for FVC and DLCO (p≥0.05). However, moderate heterogeneity was observed for FEV1/FVC ratio and TLC (p <0.05) after the application of tests of homogeneity while the heterogeneity was extremely low for decline in FEV1 and PEFR (Figure 4).

**Figure 5:**
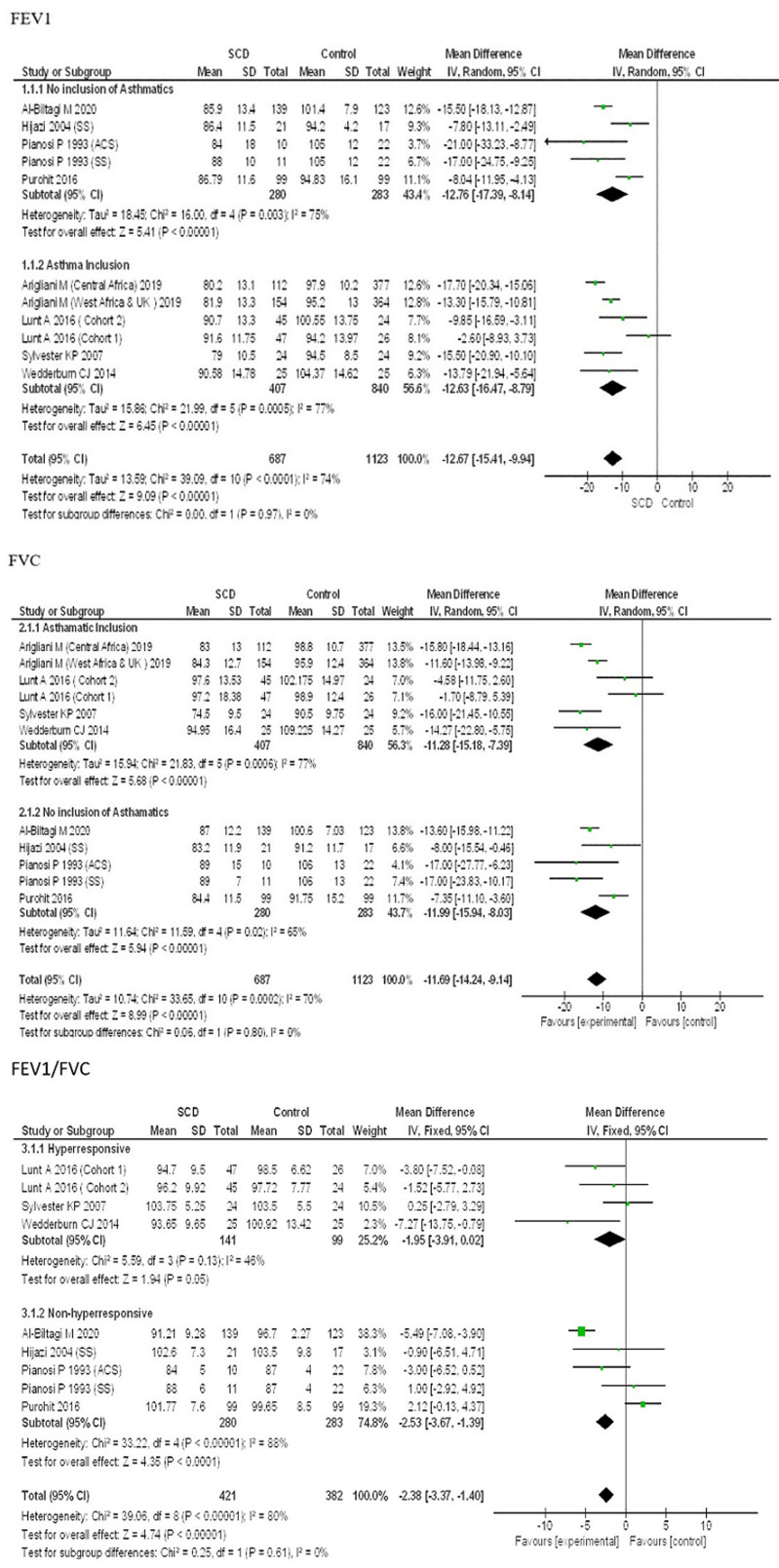
forest plot of FEV1, FVC and FEV1/FVC ratio stratified by AHR distribution

**Stratifying by AHR among subjects in the studies:** the pooled estimated mean difference for comparing SCA cases versus control for FEV1 was -12.63 (95% CI: -16.47,-8.79) for inclusion of children with AHR and -12.76 (95% CI: -17.39,-8.14) for not inclusion of children with AHR. For FVC it was -11.28 (95% CI: -15.18,-7.39) for inclusion of children with AHR and -11.99 (95% CI: -15.94,-8.03) for not inclusion of children with AHR and for FEV1/FVC it was -2.37 (95% CI: -5.19,-0.44) inclusion of children with AHR and -1.35 (95% CI: -5.10, 2.40) for not inclusion of children with AHR (subgroup differences: FEV1 P =0.97, FVC P =0.80, FEV1/FVC P =0.61) (Figure 5). Sub-group analysis suggests FEV1, FVC and FEV1/FVC ratio suggests no significant correlation for AHR in children with SCA. This indicates that heterogeneity could not be explained by this variable.

**Figure 6:**
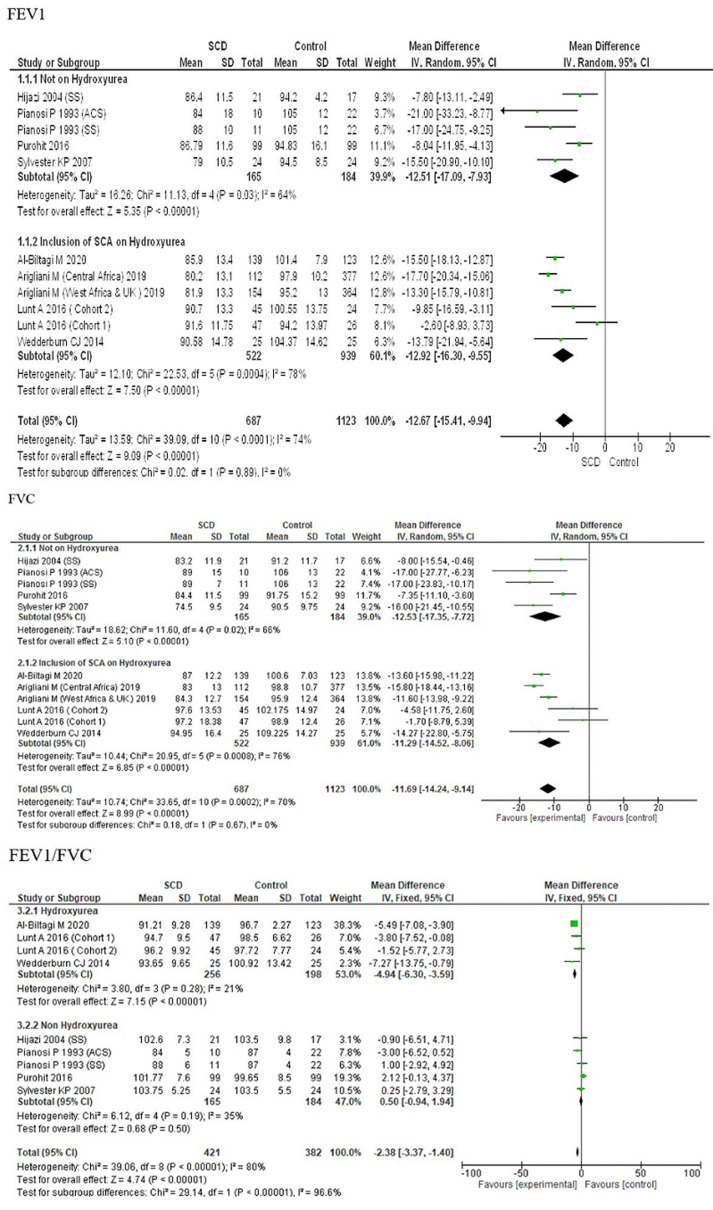
forest plots of FEV1, FVC and FEV1/FVC ratio stratified by SCA children on hydroxyurea

**Effect of hydroxyurea on PFT parameters in the studies:** the pooled estimates for FEV1 were -12.92 (95% CI: -16.30,-9.55) for inclusion of SCA children on hydroxyurea and -12.51 (95% CI: -17.09,-7.93) for exclusion of SCA children on hydroxyurea. For FVC it was -11.29 (95% CI: -14.52,-8.06) for inclusion of SCA children on hydroxyurea and -12.53 (95% CI: -17.35,-7.72) for exclusion of SCA children on hydroxyurea and for FEV1/FVC it was -4.67 (95% CI: -6.50,-2.84) inclusion of SCA children on hydroxyurea and -1.10 (95% CI: -3.23, 1.02) for exclusion of SCA children on hydroxyurea (subgroup differences: FEV1 P=0.89, FVC P=0.67, FEV1/FVC P<0.00001) (Figure 6). Sub-group analysis suggests that there was no significant correlation of hydroxyurea intake in children with SCA on changes in PFT parameters (FEV1 and FVC) with no heterogeneity. However, FEV1/FVC ratio was significantly reduced but with a very high heretogeneity.

**Figure 7:**
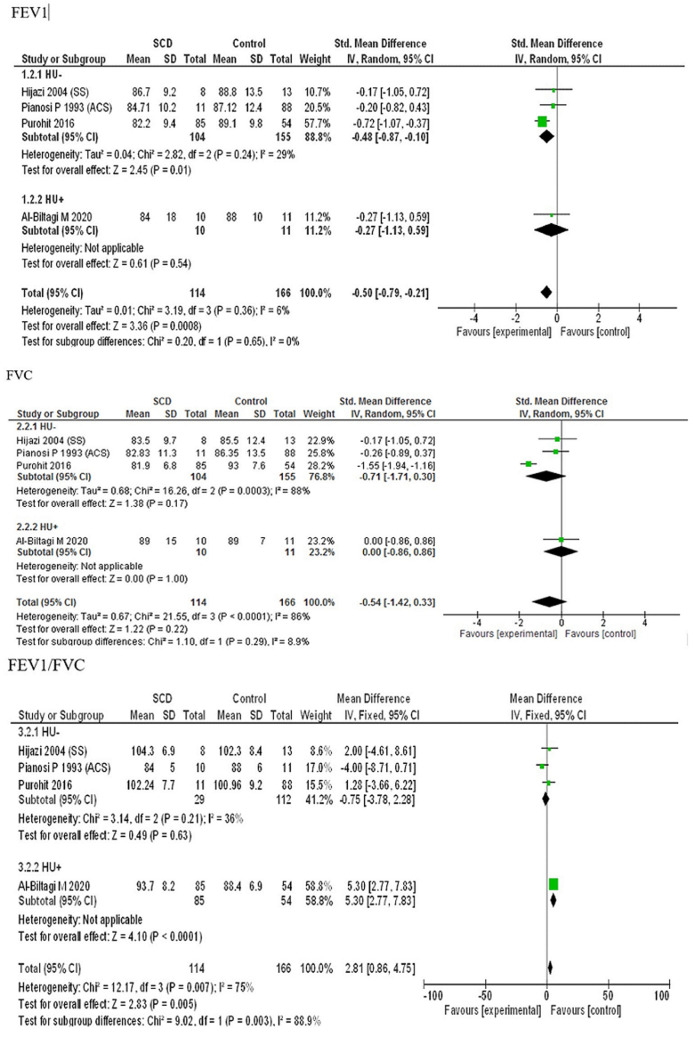
forest plots of FEV1, FVC and FEV1/FVC ratio in SCA children with ACS on hydroxyurea

**Effect of hydroxyurea on PFT parameters in children with ACS:** four studies [18-21] had included SCA children with history of ACS, with the cases being on hydroxyurea therapy in the study by Al-Biltagi *et al*. [20]. The pooled estimates for FEV1, FVC and FEV1/FVC ratio of SCA children with history of ACS on hydroxyurea therapy were -0.50 (95% CI: -0.79, -0.21; P=0.65), -0.54 (95% CI: -1.42, 0.33; P=0.29) and 2.81 (95% CI: 0.86, 4.75; P=0.003) (Figure 7). FEV1/FVC ratio was found to be significantly more in SCA children with history of ACS on hydroxyurea therapy while FEV1 and FVC were decreased, however, insignificantly (P≥0.05).

**Publication bias:** the funnel plots showed asymmetry indicative of the presence of potential publication biases (Annex 1).

## Discussion

Existing pediatric literature on PFTs of children with SCA has shown conflicting results. While obstructive type of pathology has been reported to be the most common type [21-25], few studies have shown a restrictive pattern too [20,26,27]. Among the included studies, percentage of children with obstructive pattern ranged from 4.5% to 30.9% while for the restrictive pattern ranged from 2% to 30%. This variation has brought to light the importance of pulmonary function testing in children with sickle cell anemia. To the best of our knowledge, this is the first comprehensive systematic review aimed to explore the variations in the PFT parameters seen in children with SCA. All the studies included were exclusively case-control studies. Our study analyzed the variations in all the available parameters in lung volumes in children with SCA. Our meta-analysis illustrates, that even in the absence of a pulmonary pathology such as ACS or AHR, children with SCA showed a lower lung functions (FEV1 and FVC) when compared to the normal controls, thus, implicating SCA in the progressive decline in PFT. In addition, given the high prevalence of ACS and AHR among children with SCA, it is tempting to speculate that poorly managed SCA may accelerate the progressive lung function decline. However, by means of this systematic review, it is difficult to establish causality and conclusions on progression of pulmonary decline. Conditions with increased pulmonary demands such as ACS, AHR, pulmonary infection, PH could make the child manifest this pulmonary decline clinically.

Current literature supports evidence of a relationship between obstructive lung disease and acute chest syndrome (especially recurrent episodes), particularly in the pediatric age-group [9,23,25-27]. Over a period of time, SCA is associated with chronic inflammation (ACS, AHR, recurrent chest infection, infarction, fat embolism, and smoking) that may affect the lower airways leading to the development of fibrosis and RLD; this may explain why restrictive lung disease (RLD) is more prevalent in adults than in children [28]. Children with SCA who have ACS episodes have worse pulmonary lung function than age-matched children with SCA who have not experienced ACS episodes [29]. In a retrospective analysis, Intzes *et al*. [30] found that a past history of ACS has a significant, positive correlation with obstructive lung disease but a negative, yet significant correlation with normal PFT values. Al-Biltagi *et al*. [20] also found a lower PFT parameters recording among patients who had a history of ACS in comparison to patients without ACS. Ozbek *et al*. [31] also reported a statistically significant (p <0.05) increase in % predicted values of FEV1 and FEV1/FVC ratio among children with no prior episode of ACS. The sub-group analysis of the 4 studies, which included children with previous or current episodes of ACS, showed that there was a decline in all the PFT parameters studied, except two (FVC and DLCO) where the decline was not statistically significant (P value >0.05). The significant decline in FEV1 and PEFR with extreme low heterogeneity, thus, support the role of routine FEV1 and/or PEFR in monitoring the progression of lung function decline in children with SCA with ACS.

In addition to the pathologies found in adults, children with SCA show a higher prevalence of AHR [12]. AHR among children with SCA was first evaluated by Leong *et al*. via lung volume measurements [9]. A large cohort study, based on Cooperative Study of Sickle Cell Disease (CSSCA), which followed up 291 infants for a mean period of 11 years observed 16.8% of children to be suffering from asthma [12]. Shilo *et al*. [32] reported AHR in upto 72.5% of children with SCA via methacholine challenge test (MCT) while Field *et al*. [33] reported AHR in 55% via methacholine provocation among children. Younger age, recurrent episodes of ACS, serum IgE concentration, lactate dehydrogenase (LDH) levels have been found to be associated with AHR [33,34]. The possibility of an association between AHR and ACS has also been explored in various studies. Increasing frequency of ACS episodes was associated with increased prevalence of AHR [25,34]. AHR was found to be associated with more frequent episodes of ACS (0.39 vs 0.2 events per patient per year, p <0.001). Angel *et al*. [25] also observed a significantly higher occurrence of ACS (0.44±0.44 vs 0.25±0.23, p <0.04) among asthmatic children with SCA. Although, the prevalence of AHR among children with SCA is higher than controls, this higher prevalence of AHR in SCA was found to be statistically insignificant in our meta-analysis as the decrease in PFT among SCA children with AHR for FEV1 (P=0.97), FVC (P=0.80) and FEV1/FVC ratio (P=0.48).

Hydroxyurea has evolved into a cornerstone of medical management of SCA. Hydroxyurea is a disease modifying therapy that is known to prevent and treat acute and chronic complications in SCA. As it reduces the frequency of vaso-occlusive episodes in SCA, treatment with hydroxyurea has been associated with a major 40% reduction in mortality [35]. Recent studies have shifted the focus to evaluate the additional benefits of hydroxyurea in SCA. Mclaren *et al*. [36] recorded the variations in PFT parameters in children with sickle cell disease before and after receiving hydroxyurea. A subgroup analysis of 56 HbSS patients before and after hydroxyurea initiation confirmed improved rates of decline in FEV1, FVC and forced expiratory flow between 25-75% of FVC (FEF_25-75%_) over a median period of observation. Their data suggested that hydroxyurea therapy in children with SCA led to improvement in annual PFT decline. This was credited to the favourable hematological effects of hydroxyurea in SCD. However, our meta-analysis found no statistically difference among studies where children on hydroxyurea were evaluated by PFT in comparison to studies where children had received no hydroxyurea.

**Limitations:** age-group and gender based statistics could not be derived due to the absence of uniformity of data published among the included studies. Studies which evaluated PFT parameters in terms of z-score were not be analyzed. The predominant PFT abnormality could not be ascertained as different criteria´s were used as definitions. Only a single study followed up children with abnormal lung function, therefore, the duration for worsening of PFT values could not be commented upon. The power for some subgroup analyses was limited, and relatively large heterogeneity was noted.

**Strengths:** this is the first comprehensive, quantitative analysis on the variations in PFTs in children with SCA. It includes a broad literature search, screening and data extraction performed in duplicate, a firm study quality assessment and a comprehensive data analysis, including numerous sensitivity analysis. A highly sensitive and comprehensive search of the literature was used in order to identify as many relevant studies as possible and to reduce potential publication bias. As there was no publication bias, this would extend the generalizability of the results greatly. Newcastle-Ottawa scale was used for quality assessment of the included studies. Our study re-emphasized the need for regular and routine PFTs in children with SCA for early diagnosis and better management.

**Recommendations:** in the absence of a clear consensus on PFT monitoring in children with SCA, we recommend for further analytical studies to be carried out evaluating the role of PFT for earlier detection of lung abnormalities, their impact in reducing the disease burden and improving the healthcare delivered to children with SCA. Furthermore, trials need to be carried out to study the impact of hydroxyurea on PFT as the current data available is insufficient in commenting on its efficiency.

## Conclusion

Pulmonary disease is a major cause of mortality and morbidity among children with SCA. Among the various pulmonary function test (PFT) parameters evaluated, FEV1 and FVC and were the only parameters found to be significantly decreased (P<0.05) with moderate heterogeneity in children with SCA. In comparison, there was no significant difference (P≥0.05) with high heterogeneity in the decreased values of FEV1/FVC ratio, PEFR, TLC and DLCO of children with SCA and their controls. PFT abnormalities have been found to be more common among children with frequent episodes of ACS and asthma/AHR. Pulmonary function test (PFT) measurements could, thus, be an invaluable instrument in monitoring as well as screening the individuals at high risk for pulmonary complications.

### What is known about this topic

- Pulmonary disease is a leading cause of morbidity and mortality in children with SCA;
- Both obstructive and restrictive pattern of lung functions can be seen in SCA.

### What this study adds

- Forced expiratory volume in 1 second (FEV1), forced vital capacity (FVC) showed a more significant decline in children with SCA than any other lung function parameters;
- No significant difference was seen in lung function parameters before and after the use of hydroxyurea in SCA.

## Annex

**Annex 1**: the funnel plot of: A) % predicted forced expiratory volume in 1 s (FEV1); B) % predicted forced vital capacity (FVC); C) forced expiratory volume in 1 s/forced vital capacity ratio (FEV1/FVC), [SE: standard error; MD: mean difference] (PDF-151KB)
